# A meta-analysis of the diagnostic value of microRNA for hypertensive left ventricular hypertrophy

**DOI:** 10.3389/fcvm.2022.994826

**Published:** 2022-10-26

**Authors:** Su-Hai Fei, Zhen-Feng Liu, Hai-Ning Xie, Jia-Ni Tong, Zheng-Mei Fang, Yan Chen, Ying-Shui Yao

**Affiliations:** ^1^School of Public Health, Wannan Medical College, Wuhu, China; ^2^Wannan Medical College, Institute of Chronic Disease Prevention and Control, Wuhu, China; ^3^Department of Clinical Medicine, Anhui College of Traditional Chinese Medicine, Wuhu, China

**Keywords:** hypertension, left ventricular hypertrophy, diagnosis, microRNA, meta-analysis

## Abstract

In this study, the diagnostic value of microRNAs (miRNAs) for hypertension (HTN) with left ventricular hypertrophy (LVH) were evaluated by meta-analysis. A correlation study of the diagnostic value of miRNAs in HTN with LVH was conducted using a computer search of the China Knowledge Network (CNKI), Wanfang, VIP, China Biomedical Literature Database (CBM), PubMed, Web of Science, and Embase. Studies from the time of database creation to May 2022 were evaluated. The quality assessment of diagnostic accuracy studies-2 (QUADAS-2) tool in RevMan 5.3 was used to evaluate the quality of the literature, and Meta-Disc 1.4 and Stata 16.0, were used to calculate the combined sensitivity (SEN), specificity (SPE), positive likelihood ratio (PLR), negative likelihood ratio (NLR), diagnostic advantage ratio (DOR), and their 95% confidence intervals. Subject working characteristic curves were plotted and the area under the curve (AUC) was calculated using Stata 16.0. Seven publications and 8 studies were included. miRNA diagnoses of HTN with LVH had SEN_combined_ = 0.84, SPE_combined_ = 0.80, PLR_combined_ = 4.2, NLR_combined_ = 0.20, DOR_combined_ = 21, and AUC_combined_ = 0.89. Subgroup analysis showed that the sensitivity of plasma miRNA for the diagnosis of HTN with LVH was 0.85, which was higher than that of serum which was 0.83. The specificity of serum miRNA for the diagnosis of HTN with LVH was 0.82, which was higher than that of plasma which was 0.78, and the diagnostic accuracy of miRNA in serum DOR was 23, which was higher than that of plasma DOR which was 20. In the diagnosis of HTN with LVH, miRNA has high sensitivity and specificity and is a better biological marker.

**Systematic review registration:**

http://www.crd.york.ac.uk/PROSPERO/, CRD42022346686.

## Introduction

Hypertension (HTN) is a systemic chronic disease that primarily manifests as elevated arterial blood pressure in the circulatory system. Chronic poor blood pressure control can cause changes in the structure and function of the heart and can lead to left ventricular hypertrophy (LVH).

Cardiovascular disease (CVD) is one of the leading causes of death worldwide, particularly in developed countries. In 2019, the global prevalence of CVD reached 523 million cases, resulting in 18.6 million deaths (1). HTN and LVH are both independent risk factors for CVD (2). Studies have shown that the incidence of LVH in patients with HTN ranges from 10 – 40% (3). The weighted risk of HTN combined with LVH exacerbates the occurrence and progression of CVD. LVH is an important target organ in hypertensive pathological damage and LVH is a major cause of heart failure and cardiovascular death in patients with HTN (4). Therefore, early detection and diagnosis of HTN with LVH are particularly important for early treatment to reverse the formation of an LVH lesion.

Currently, the clinical diagnosis of LVH in patients with HTN relies on electrocardiography (ECG), echocardiography (ECHO), and cardiac magnetic resonance imaging (MRI) (5). ECG is simple and easy to perform, but the ventricular muscle group, chest wall thickness, and cavity location affect the effectivity of the examination. The diagnostic sensitivities of ECG for mild and moderate/severe LVH are 7–35% and 30–60%, respectively (6). Cardiac MRI has higher sensitivity and specificity, but is time-consuming and expensive. ECHO is currently the most commonly used clinical method, and its sensitivity for diagnosing LVH is higher than that of ECG (7). However, the accuracy of ECHO in measuring LVH is linked to the physician's choice of 2D ultrasound views, which is highly subjective, and leads to the measurement results varying from physician to physician. The process of obtaining diagnostic indicators requires basic patient information and complex calculations, resulting in inconvenience in clinical diagnostic operations (8). Therefore, it is important to develop a simple test method with high sensitivity and specificity. Recently, researchers have conducted numerous studies on the pathogenesis associated with cardiac hypertrophy. Although the detailed mechanisms are unclear, the role of microRNAs (miRNAs) in the cardiovascular field is gaining increasing attention (9).

miRNAs are a class of small non-coding RNAs that are 18–25 nucleotides in length (10). miRNAs are involved in the physiopathogenesis of CVD and have important regulatory roles in myocardial remodeling, proliferation, and differentiation, and their abnormal expression or function can mediate pathological myocardial hypertrophy (11). miRNAs are associated with HTN and LVH, and differences in expression have been observed in clinical studies and studies of regulatory mechanisms in animal models. miR-1 is expressed in the myocardium and negatively regulates myocardial hypertrophy (12). Wang et al. demonstrated through animal studies that miR-195 expression in the cardiac tissue of rats with hypertension combined with myocardial hypertrophy was approximately four times higher than that in rats without myocardial hypertrophy (13). Wang et al. showed that miR-27b overexpression promoted cardiomyocyte hypertrophy *in vitro* and that inhibition of miR-27b expression improved norepinephrine-induced cardiomyocyte hypertrophy (14). This suggests that miRNA may be a potential diagnostic and therapeutic target for HTN with LVH. Recently, several studies have explored miRNAs as diagnostic markers in the peripheral blood of patients with HTN and LVH.

In this study, the diagnostic efficacy of miRNAs in patients with HTN and LVH was systematically evaluated using meta-analysis to provide a reference for clinical practice and application.

## Materials and methods

### Literature search strategy

Correlation studies of miRNA on the diagnostic value of LVH in HTN were collected by searching the following databases: the China Knowledge Network (CNKI), Wanfang, VIP, China Biomedical Literature Database (CBM), PubMed, Web of Science, and Embase. Studies from the time of database creation to May 2022 were evaluated. The search terms included: miRNA, hypertension, and left ventricular hypertrophy.

### Inclusion and exclusion criteria

The inclusion criteria were as follows: (1) the study was conducted in humans, (2) the study was a case-control study, (3) the study investigated the diagnostic value of miRNAs for HTN with LVH, (4) the study provided sufficient data to satisfy the construction of a 2 × 2 four-compartment table, and (5) the control group was HTN without LVH.

The exclusion criteria were as follows: (1) non-human experiments; (2) trials without control groups; (3) healthy individuals in the control group; (4) reviews, abstracts, and conference proceedings; (5) repeated published literature; and (6) lack of access to the original text and data.

### Data abstraction and quality assessment

Two investigators were screened independently, and data extraction was performed based on the inclusion and exclusion criteria after careful reading of the full text. The extracted data included the first author, year of publication, country, case group, control group, miRNA, sample source, assay, true positive (TP), false positive (FP), false negative (FN), and true negative (TN) results. In the process of literature screening, data extraction, and quality assessment, any disagreements were resolved in consultation with the third investigator.

The quality assessment of diagnostic accuracy studies-2 (QUADAS-2) tool was applied to evaluate the quality of the literature, including the case selection, trials to be evaluated, gold standard, case flow, and progress (15).

### Statistical analysis

Data were statistically analyzed using RevMan 5.3, Stata 16.0, and Meta-Disc 1.4 software. The combined sensitivity, specificity, positive likelihood ratio, negative likelihood ratio, diagnostic advantage ratio, and their respective 95% confidence intervals (CIs) were calculated, and summary receiver operating characteristic (SROC) curves were plotted to calculate the area under the curve (AUC). Heterogeneity was assessed using the Q statistic and *I*^2^ test; if the test result was *P* > 0.05, (Q statistic) and *I*^2^ < 50%, then the included studies were homogeneous, and the combined statistic was calculated using the fixed-effects model. If *P* < 0.05 or *I*^2^ > 50%, a random-effects model was used. In the case of heterogeneity, the size of the threshold effect was assessed using the Spearman rank correlation coefficient between the log of sensitivity and the log of (1-specificity) using Meta-Disc 1.4 software. The Deek's test was used to assess publication bias of the included studies. Statistical significance was set at *P* < 0.05.

## Results

### Included studies

**Figure 1:**
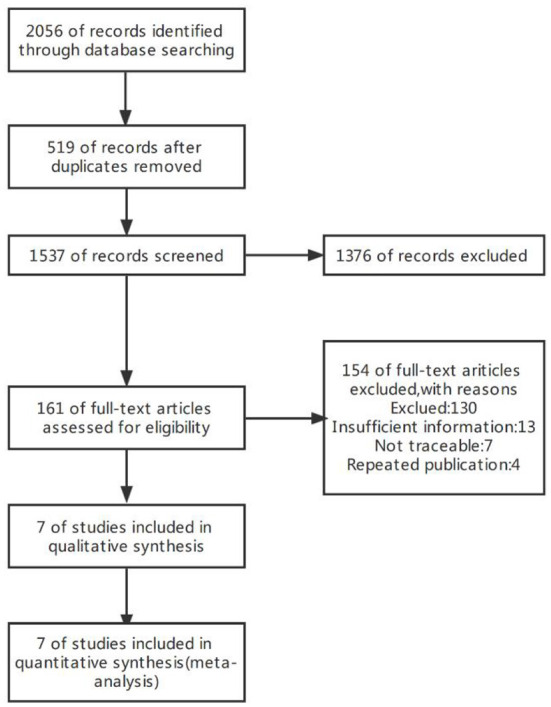
Flow diagram of literature search and exclusion process.

The initial search yielded 2,056 publications, of which 519 duplicate publications were excluded. After reading the titles and abstracts, 1,376 studies which clearly did not meet the inclusion criteria were excluded. After reading the full text, 154 articles were excluded according to the exclusion criteria, and seven studies were finally included (Figure 1).

### Characteristics of the included studies

**Figure 2:**
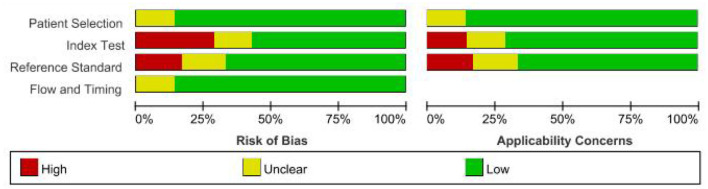
Bar chart of QUADAS-2 risk of bias evaluation.

Seven articles (16–22) and eight diagnostic accuracy studies were included. A total of 1,322 study subjects were included, including 679 cases and 643 controls (Table 1). The QUADAS-2 tool was used to evaluate the quality of the studies (Figure 2), which included case selection, trials to be evaluated, gold standard, case flow, and progress.

**Table 1:** The characteristics of the included studies.

**Author**	**Year**	**Country**	**N cases/control**	**miRNA**	**Sample**	**Test method**	**TP**	**FP**	**FN**	**TN**
Wu et al. (16)	2020	China	35/87	miR-21	Serum	RNA Extraction Kit and PCR	26	7	9	80
Zhou et al. (17)	2015	China	80/80	miR-29b	Serum	qRT-PCR	75	11	5	69
Wang et al. (18)	2015	China	38/56	miR-27b	Plasma	qRT-PCR	28	11	10	45
Deng et al. (19)	2016	China	118/108	miR-1	Serum	RNA Extraction Kit and PCR	95	28	23	80
				miR-195						
				miR-27b						
Lin et al. (20)	2016	China	70/70	miR-1	Plasma	qRT-PCR	66	7	4	63
Wu et al. (21)	2014	China	69/71	miR-26b	Plasma	qRT-PCR	56	20	13	51
Wu et al. (22)	2014	China	69/71	miR-30	Plasma	qRT-PCR	58	24	11	47
Wang et al. (24)	2015	China	200/100	miR-27b	Serum	qRT-PCR	158	30	42	70

TP, true positive; FP, false positive; FN, false negative; TN, true negative.

### Heterogeneity test

The sensitivity (SEN) and specificity (SPE) I^2^ values were 71.33 and 79.07%, respectively, calculated using Stata 16.0. There was large heterogeneity among the included studies. Spearman rank correlation analysis was performed using the SEN logarithm with the 1-SPE logarithm (*r* = 0.000, *P* = 1.000). ROC curves were plotted and did not show a shoulder-arm distribution, suggesting no threshold effect.

### Meta-analysis and ROC curves

**Figure 3:**
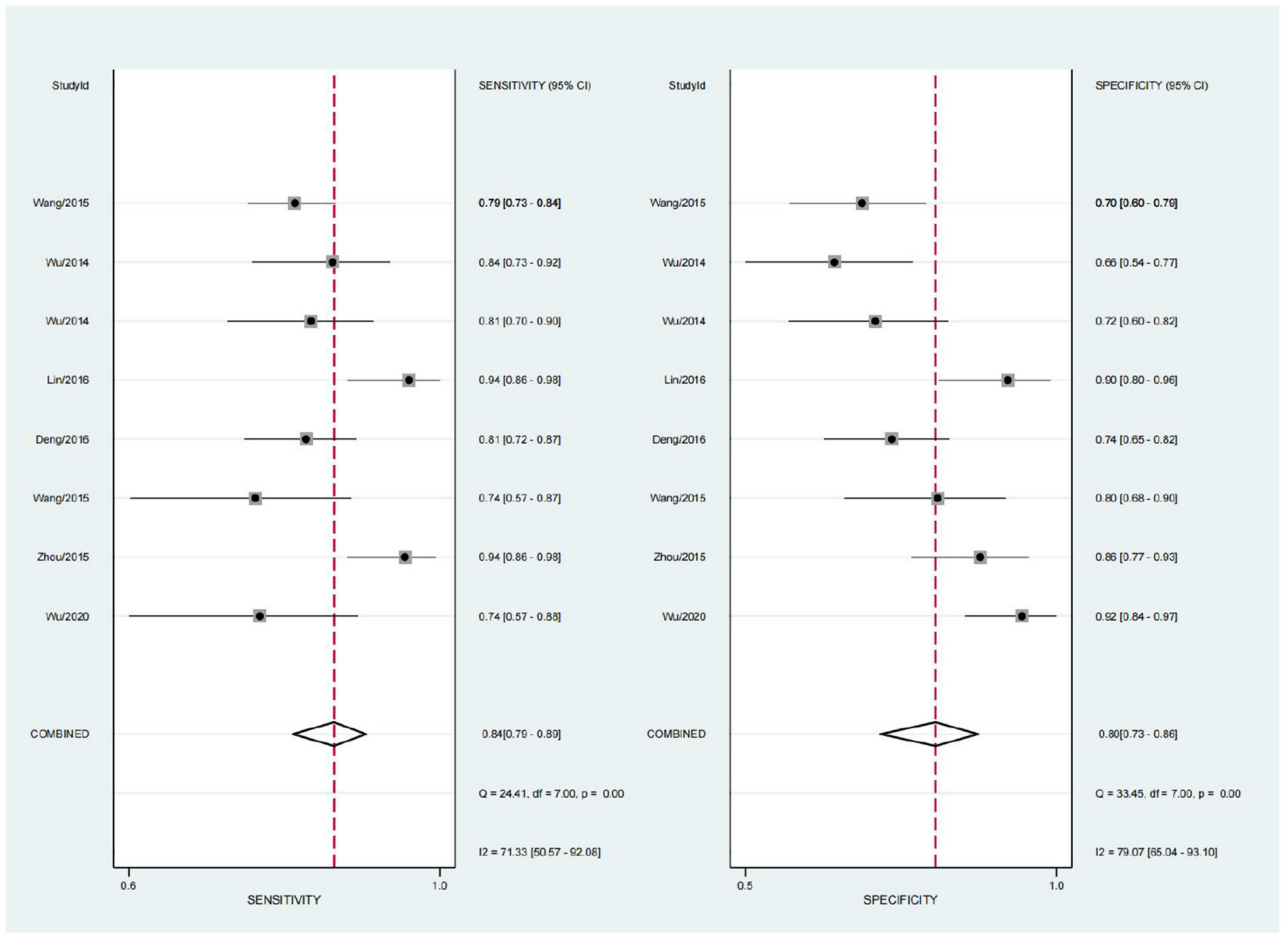
Combined sensitivity and specificity of miRNA in the diagnosis of HTN with LVH.

**Figure 4:**
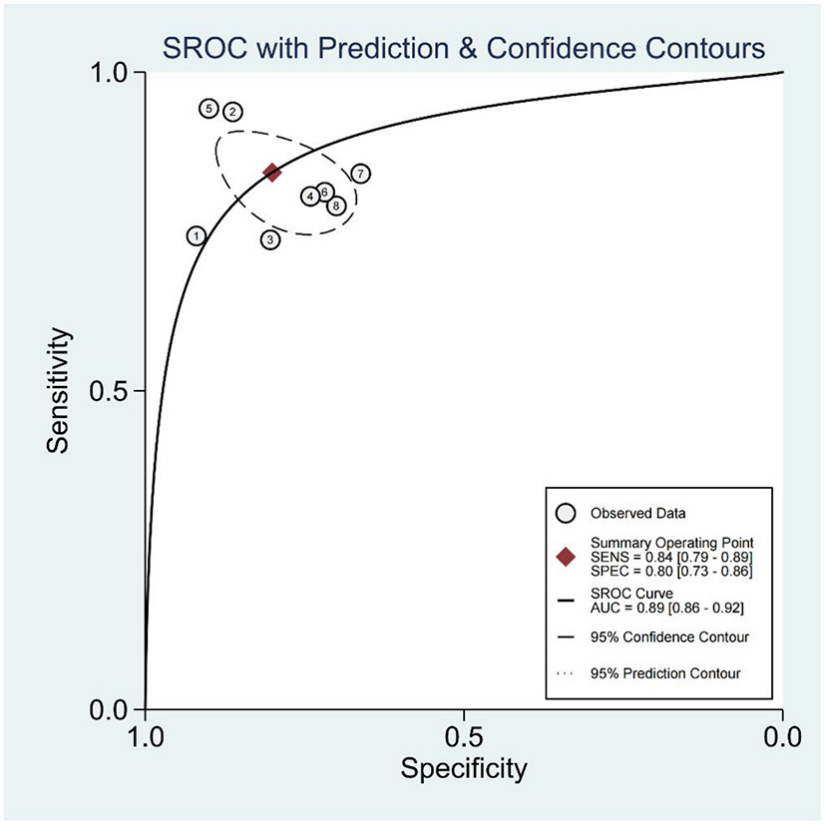
ROC curve of miRNA for diagnosis of HTN with LVH.

A random effects model was used for merging, the SEN_combined_ = 0.84 (95% CI; 0.79, 0.89), SPE_combined_ = 0.80 (95% CI; 0.73, 0.86) for miRNA diagnosis of HTN with LVH (Figure 3), PLR_combined_ = 4.2 (95% CI; 2.9, 6.1), NLR_combined_ = 0.20 (95% CI; 0.14, 0.28), DOR_combined_ = 21 (95% CI; 11, 42), and AUC_combined_ = 0.89 (95% CI; 0.86, 0.92) (Figure 4). The above results suggest that miRNAs have good diagnostic value for HTN with LVH.

### Subgroup analysis

Subgroup analyses were performed based on the sources of the samples. The sensitivity of miRNA in plasma for the diagnosis of HTN with LVH was 0.85 (95% CI; 0.77, 0.91), which was higher than that of serum which was 0.83 (95% CI; 0.75, 0.89). The specificity of miRNA in serum for the diagnosis of HTN with LVH was 0.82 (95% CI; 0.71, 0.89), which was higher than that of plasma which was 0.78 (95% CI; 0.67, 0.86). The diagnostic accuracy of miRNA in serum diagnostic advantage ratio (DOR) was 23 (95% CI; 9, 55), which was higher than that of plasma DOR which was 20 (95% CI; 7, 57) (Table 2).

**Table 2:** Subgroup analysis.

	**SEN**	**SPE**	**PLR**	**NLR**	**DOR**
**Sample source**					
Serum	0.83 (0.75, 0.89)	0.82 (0.71, 0.89)	4.60 (2.70, 7.70)	0.20 (0.13, 0.32)	23 (9, 55)
Plasma	0.85 (0.77, 0.91)	0.78 (0.67, 0.86)	3.90 (2.30, 6.40)	0.19 (0.11, 0.34)	20 (7, 57)

SEN, sensitivity; SPE, specificity; PLR, positive likelihood ratio; NLR, negative likelihood ratio; DOR, diagnostic advantage ratio.

### Fagan nomogram analysis

**Figure 5:**
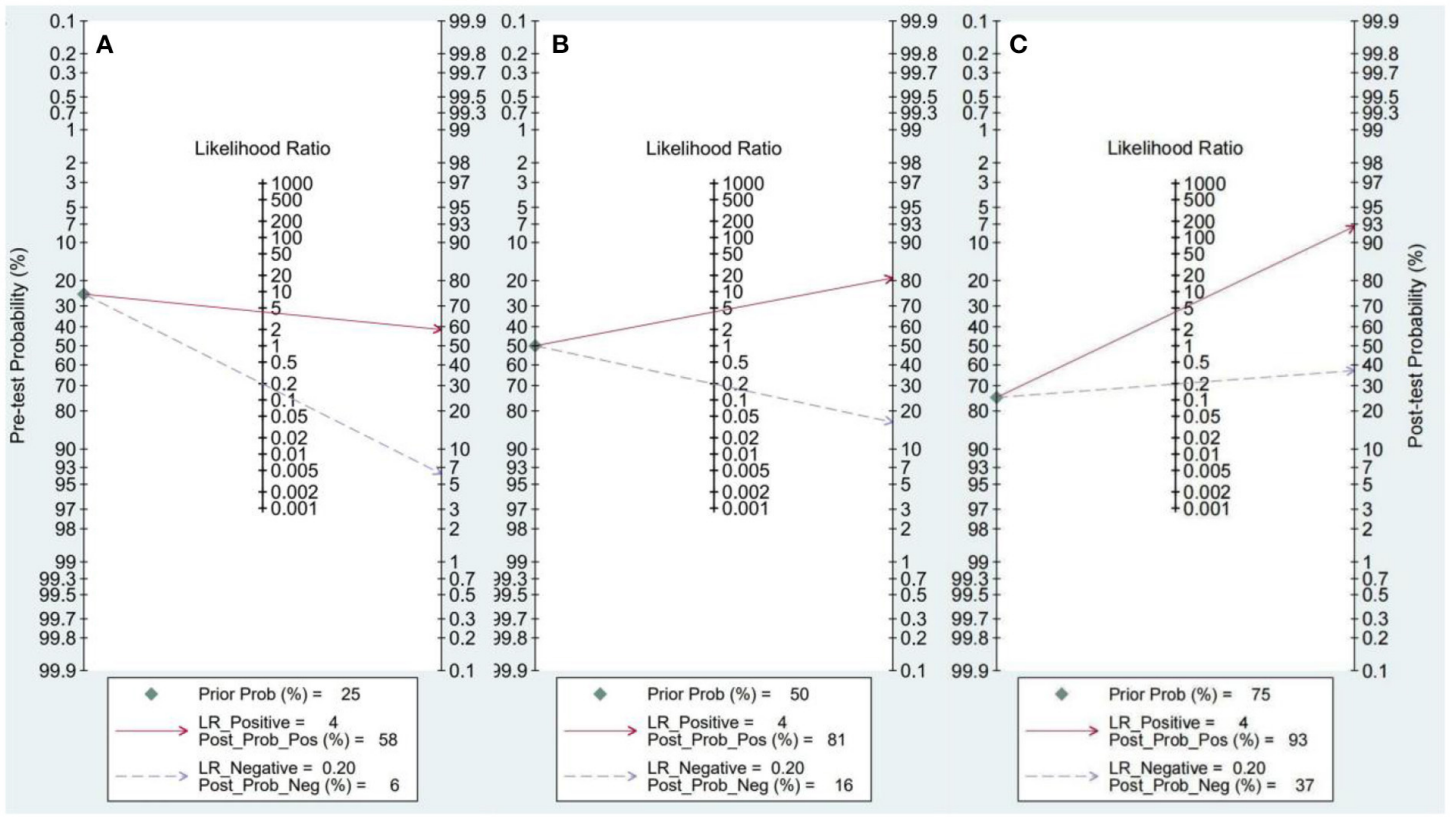
Fagan nomogram. **(A)** 25% probability of disease before test. **(B)** 50% probability of disease before test. **(C)** 75% probability of disease before test.

The Fagan nomogram analysis showed that for patients with a 25% pre-test probability of having HTN with LVH, the probability of having HTN with LVH was 58% when the miRNA test was positive, and the post-test probability was reduced to 6% when the miRNA test was negative. Patients with a 50% pre-test probability of developing HTN with LVH exhibited an 81% probability of developing HTN with LVH when the miRNA test result was positive and 16% when the miRNA test result was negative. Patients with a 75% pre-test probability of having HTN with LVH exhibited a 93% probability of having HTN with LVH when the miRNA test was positive and 37% when the miRNA test was negative (Figure 5).

### Sensitivity analysis

Sensitivity analysis using an exclusion method that excluded studies individually showed no significant changes in combined sensitivity, specificity, or diagnostic advantage ratio. This suggested that the meta-analysis results were more robust.

### Publication bias

**Figure 6:**
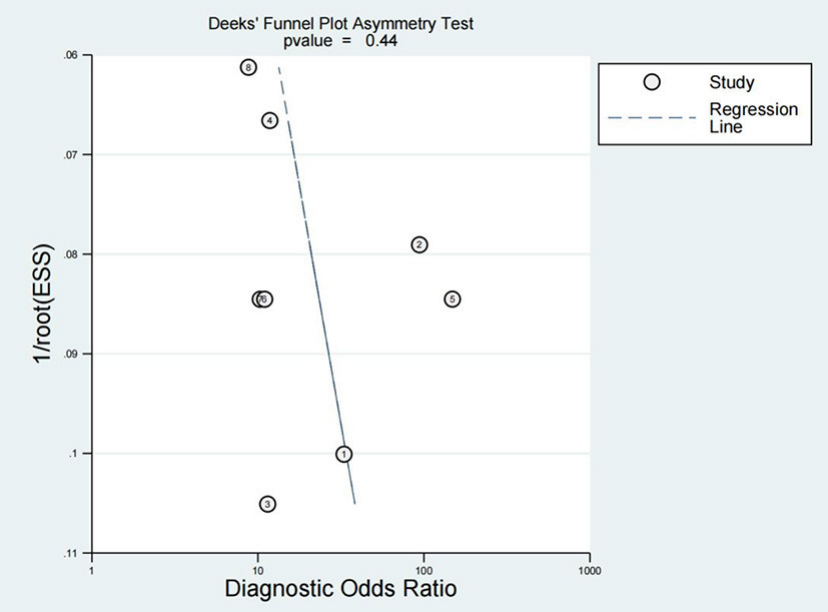
Deeks funnel diagram.

Publication bias is considered an additional factor affecting diagnostic accuracy. Deek's funnel plot was produced using Stata 16.0, and the inclusion of seven studies was evenly distributed on both sides of the regression line (*P* = 0.44), suggesting no significant publication bias (Figure 6).

## Discussion

HTN is a risk factor for several CVDs, and can lead to organ damage and dysfunction, placing a significant load on the ejection function of the heart. Long-term HTN increases the LV load and causes changes in LV morphology, structure, and function, leading to LVH (23). The prevalence and mortality of HTN with LVH worldwide are increasing yearly (24). The development of LVH often leads to adverse prognostic outcomes such as heart failure and sudden death; many patients fail to be diagnosed in the early stages of LVH, which results in losing the best time to intervene, leading to disease. This leads to irreversible progression of the disease. Therefore, early diagnosis of LVH is important. Currently, ECHO is used to diagnose LVH (25), but the detection rate is unsatisfactory.

Therefore, it is important to identify other simple methods to improve the detection rate. Blood biomarker tests are non-invasive, inexpensive, and clinically feasible. Although miRNAs account for <1% of coding genes, more than one-third of the human genes are controlled by miRNAs (26). miRNAs play an important role in cardiac diseases such as coronary heart disease and myocarditis (27). They can regulate gene expression by targeting mRNAs for degradation or directly inhibiting the translation process to affect the physiological and pathological processes of the heart (28). Studies have confirmed that miRNAs are involved in the regulation of cardiac remodeling, coronary vasculopathy, arrhythmias, and other cardiovascular disease processes, especially in the development of myocardial hypertrophy (29). miRNAs have many advantages over traditional markers, such as easy degradation, more reliable measurement of expression levels, more stable human samples, more rapid collection, and less invasiveness (30).

To systematically evaluate the diagnostic value of circulating miRNA expression for HTN with LVH, this study comprehensively searched relevant literature, and after strict implementation of the inclusion and exclusion criteria, a total of seven papers with 1,322 study subjects were included. The diagnosis of HTN with LVH using miRNA showed a result of SEN = 0.84 and SPE = 0.80, indicating a 16% under diagnosis rate and a 20% misdiagnosis rate. The positive likelihood ratio (PLR) was 4.2, suggesting that the probability of a positive miRNA test in the HTN with LVH population was four times higher than the probability of a positive test in the normal population. The negative likelihood ratio (NLR) was 0.20, suggesting that 20% of those with a negative miRNA test result may be HTN with LVH. The DOR can be used to determine the degree of association between the results of a diagnostic test and the disease. When the DOR is >1, the larger the value, the higher the discriminatory accuracy of the diagnostic test (31). In this study, the DOR value was 21, suggesting that miRNAs have a high ability to accurately identify high HTN with LVH. The AUC value was 0.89, indicating a high diagnostic efficacy. In the subgroup analysis, the diagnostic accuracy of miRNA in serum DOR was 23 (95% CI; 9, 55), which was higher than that of plasma DOR which was 20 (95% CI; 7, 57).

This study had some shortcomings: (1) Studies of miRNA tests for HTN with LVH only started in recent years, and there are few related reports. Although multiple paths were taken to collect domestic and international literature, language limitations led to only Chinese and English literature being analyzed in this study. This may have resulted in a lack of literature. (2) There may have been some population bias in this study, and the combined effect sizes were all based on Chinese people. (3) The studies exhibited heterogeneity due to the different researchers in different studies, time and method of sample processing and storage, instruments and methods of collecting miRNA, and severity of HTN with LVH in patients. This affected the accuracy of the meta-analysis results. In future diagnostic trials, the selection criteria of study subjects should be clarified, and blinding of the trial process should be ensured. (4) The large heterogeneity among studies may be caused by the different cut-off value settings of the included studies and the different types of miRNAs; however, the limitation of the number of studies prevents in-depth subgroup analysis. Therefore, well-designed, high-quality, prospective studies with large sample sizes and long-term follow-up are required to accurately reflect the diagnostic efficacy of miRNAs.

## Conclusion

The diagnosis of HTN with LVH using miRNAs exhibits high sensitivity and specificity and shows that miRNAs are better biological markers. However, the limitations listed above mean that the above conclusions need to be validated by more high-quality studies.

## Data availability statement

The original contributions presented in the study are included in the article/supplementary material, further inquiries can be directed to the corresponding author/s.

## Author contributions

This study was designed by S-HF. S-HF and Z-FL contributed data to the paper. Statistical analysis and interpretation of data were performed by S-HF, Z-FL, and H-NX. All authors were involved in drafting and revision of the manuscript for important intellectual content and approved the final version to be published.

## Funding

This work was supported by the Fifth Batch of Talents Selected under the Special Support Plan in Anhui Province (Organization Department of Anhui Provincial Party Committee [2019] No. 14, T000516), National Natural Science Foundation of China (81874280 and 81673266), and Major Project of Natural Science Research in Anhui Universities (KJ2021ZD0098).

## Conflict of interest

The authors declare that the research was conducted in the absence of any commercial or financial relationships that could be construed as a potential conflict of interest.

## Publisher's note

All claims expressed in this article are solely those of the authors and do not necessarily represent those of their affiliated organizations, or those of the publisher, the editors and the reviewers. Any product that may be evaluated in this article, or claim that may be made by its manufacturer, is not guaranteed or endorsed by the publisher.

## References

[1] Roth GA Mensah GA Johnson CO Addolorato G Ammirati E Baddour LM . Global burden of cardiovascular diseases and risk factors, 1990–2019: update from the GBD 2019 study. J Am Coll Cardiol. (2020) 76:2982–3021. 10.1016/j.jacc.2020.11.010 33309175 PMC7755038

[2] Devereux RB. Therapeutic options in minimizing left ventricular hypertrophy. Am Heart J. (2000) 139:S9–14. 10.1067/mhj.2000.102902 10618582

[3] Umemura S Arima H Arima S Asayama K Dohi Y Hirooka Y . The Japanese society of hypertension guidelines for the management of hypertension (JSH 2019). Hypertens Res. (2019) 42:1235–481. 10.1038/s41440-019-0284-9 31375757

[4] Mancia G Fagard R Narkiewicz K Redon J Zanchetti A Böhm M . 2013 ESH/ESC guidelines for the management of arterial hypertension: the task force for the management of arterial hypertension of the european society of hypertension (ESH) and of the European society of cardiology (ESC). Eur Heart J. (2013) 34:2159–219. 10.1093/eurheartj/eht151 23771844

[5] Dunn FG Pfeffer MA. Left ventricular hypertrophy in hypertension. N Engl J Med. (1999) 340:1279–80. 10.1056/nejm199904223401610 10210713

[6] Murphy ML Thenabadu PN De Soyza N Meade J Doherty JE Baker BJ. Sensitivity of electrocardiographic criteria for left ventricular hypertrophy according to type of cardiac disease. Am J Cardiol. (1985) 55:545–9. 10.1016/0002-9149(85)90244-9 3155902

[7] Cuspidi C Sala C Negri F Mancia G Morganti A. Prevalence of left-ventricular hypertrophy in hypertension: an updated review of echocardiographic studies. J Hum Hypertens. (2012) 26:343–9. 10.1038/jhh.2011.104 22113443

[8] Chen M Bu F. A comparative study of electrocardiography and echocardiography in the diagnosis of left ventricular hypertrophy in patients with essential hypertension. Dis Surveill Control. (2015) 9:875–7.

[9] Barwari T Joshi A Mayr M. MicroRNAs in Cardiovascular Disease. J Am Coll Cardiol. (2016) 68:2577–84. 10.1016/j.jacc.2016.09.945 27931616

[10] Li W Zhang H Wang Y Dong J Li D Jin X. Research progress of microRNA in cardiovascular disease. Chin Med Sci. (2021) 11:55–7. 10.3969/j.issn.2095-0616.2021.17.01435899516

[11] Duan L Xiong X Liu Y Wang J. microRNA and left ventricular hypertrophy Chinese Journal of Traditional Chinese Medicine. (2014) 39:3211–5. 25522599

[12] Seok H Lee H Lee S Ahn SH Lee HS Kim GD . Position-specific oxidation of miR-1 encodes cardiac hypertrophy. Nature. (2020) 584:279–85. 10.1038/s41586-020-2586-0 32760005

[13] Wang W Luo Y Hong X. The role of MicroRNA-195 and TGF-β1/Smads signaling pathway in cardiac remodeling in spontaneously hypertensive rats. Chin J Arteriosc. (2014) 22:121–6.

[14] Wang J Song Y Zhang Y Xiao H Sun Q Hou N . Cardiomyocyte overexpression of miR-27b induces cardiac hypertrophy and dysfunction in mice. Cell Res. (2012) 22:516–27. 10.1038/cr.2011.132 21844895 PMC3292295

[15] Whiting PF Rutjes AW Westwood ME Mallett S Deeks JJ Reitsma JB . QUADAS-2: a revised tool for the quality assessment of diagnostic accuracy studies. Ann Intern Med. (2011) 155:529–36. 10.7326/0003-4819-155-8-201110180-00009 22007046

[16] Wu L Gao J Zhang S Xia J. Expression and clinical significance of serum miR-21 and TGF-β 1 in hypertensive patients with left ventricular hypertrophy. J Lab Med. (2021) 42:2482–6. 10.3969/j.issn.1673-4130.2021.20.011

[17] Zhou X Cai J Liu W Zhang X Wu X Gao C. The value of circulating microRNA-29b in the diagnosis of hypertensive left ventricular hypertrophy. Chin J Int Med. (2019) 34:278–81. 10.3760/cma.j.issn.0578-1426.2019.04.008 30917420

[18] Wang G. Study on the Diagnostic Value of miR-27b in Exosomes for Hypertensive Left Ventricular Hypertrophy. Guangzhou: Guangzhou Medical University (2017).

[19] Deng B Ye S Song B & Lu Q. The relationship between serum microRNA-1, 27b and 195 levels and combined detection in patients with hypertension and left ventricular hypertrophy and disease assessment and prognosis. Chin J Hypert. (2020) 28:1213–7. 10.16439/j.cnki.1673-7245.2020.12.028

[20] Lin D Zhong J Zheng Y Zhang Y. The value of miRNA-1 in the diagnosis of hypertensive left ventricular hypertrophy. J Clin Cardiovas Disease. (2017) 33:962–5. 10.13201/j.issn.1001-1439.2017.010.011

[21] Wu C. The Diagnostic Value of plasma miR-26b and miR-30a in hypertensive Left Ventricular Hypertrophy and Hypertrophic Cardiomyopathy. Guangzhou: Guangzhou Medical University (2015).

[22] Wang Y Chen S Gao Y Zhang S. Serum microRNA-27b as a screening biomarker for left ventricular hypertrophy. Tex Heart Inst J. (2017) 44:385–9. 10.14503/thij-16-5955 29276436 PMC5737148

[23] Shenasa M. & Shenasa H. Hypertension, left ventricular hypertrophy, and sudden cardiac death Int J Cardiol. (2017) 237:60–3. 10.1016/j.ijcard.2017.03.002 28285801

[24] Korner PI Jennings GL. Assessment of prevalence of left ventricular hypertrophy in hypertension. J Hypertens. (1998) 16:715–23. 10.1097/00004872-199816060-00001 9663910

[25] Zhang W Zhou Y Bai B Yu S Xiong J Chi C . Consistency of left ventricular hypertrophy diagnosed by electrocardiography and echocardiography: the Northern Shanghai study. Clin Interv Aging. (2019) 14:549–56. 10.2147/cia.S180723 30880935 PMC6417007

[26] Krol J Loedige I Filipowicz W. The widespread regulation of microRNA biogenesis, function and decay. Nat Rev Genet. (2010) 11:597–610. 10.1038/nrg2843 20661255

[27] Thum T Catalucci D Bauersachs J. MicroRNAs: novel regulators in cardiac development and disease. Cardiovasc Res. (2008) 79:562–70. 10.1093/cvr/cvn137 18511432

[28] Correia De Sousa M Gjorgjieva M Dolicka D Sobolewski C Foti M. Deciphering miRNAs' Action through miRNA Editing. Int J Mol Sci. (2019) 20:6249. 10.3390/ijms20246249 31835747 PMC6941098

[29] Tiwari A Mukherjee B Dixit M. MicroRNA key to angiogenesis regulation: MiRNA biology and therapy. Curr Cancer Drug Targets. (2018) 18:266–77. 10.2174/1568009617666170630142725 28669338

[30] Chen X Ba Y Ma L Cai X Yin Y Wang K . Characterization of microRNAs in serum: a novel class of biomarkers for diagnosis of cancer and other diseases. Cell Res. (2008) 18:997–1006. 10.1038/cr.2008.282 18766170

[31] Tong F Chen K He H. Application of odds ratio in diagnostic test evaluation. Chin J Epidemiol. (2005) 3:89–90. 10.3760/j.issn:0254-6450.2005.10.020

